# A Bayesian approach to predict performance in football: a case study

**DOI:** 10.3389/fspor.2025.1486928

**Published:** 2025-03-14

**Authors:** Gabriel G. Ribeiro, Lilia C. C. da Costa, Paulo H. Ferreira, Diego C. do Nascimento

**Affiliations:** ^1^ Department of Statistics, Federal University of Bahia, Salvador, Brazil; ^2^ Departamento de Matemática, Facultad de Ingeniería, Universidad de Atacama, Copiapó, Chile

**Keywords:** Bayesian inference, dynamic models, football prediction, quantitative football performance, dynamic zero-inflated Poisson

## Abstract

Football is the most practiced sport in the world and can be said to be unpredictable, i.e., it sometimes presents surprising results, such as a weaker team overcoming a stronger one. As an illustration, the Brazilian Championship Series A (*Brasileirão*) has historically been shown to be one of the most outstanding examples of this unpredictability, presenting a large number of unexpected outcomes (perhaps given its high competitiveness). This study unraveled attack and defense patterns that may help predict match results for the 2022 Brazilian Championship Series A, using data-driven models considering 10 variations of the Poisson countable regression model (including hierarchy, overdispersion, time-varying parameters, or informative priors). As informative priors, the 2021 Brazilian Championship Series A’s information from the previous season was adopted for each team’s attack and defense advantage estimations. The proposed methodology is not only helpful for match prediction but also beneficial for quantifying each team’s attack and defense dynamic performances. To assess the quality of the forecasts, the de Finetti measure was used, in addition to comparing the goodness-of-fit using the leave-one-out cross-validation metric, in which the models presented satisfactory results. According to most of the metrics used to compare the methods, the dynamic Poisson model with zero inflation provided the best results, and, to the best of our knowledge, this is the first time this model has been used in a subjective football match context. An online framework was developed, providing interactive access to the results obtained in this study in a Shiny app.

## 1 Introduction

Football is the world’s most popular sport and it has deep historical roots tracing back to ancient civilizations (1). The game evolved from a simple ball-kicking activity to an organized sport (2). Moreover, historically, football has shown a slow evolution of different ball games that took place over thousands of years, from the simplest and most rudimentary forms to the most complex structures currently observed (1). In addition, regardless of football’s stochasticity, many studies have shown the utility of the application of mathematics in match predictability and quantification of a team’s attack–defense performance (3, 4).

Football is defined by its unpredictability. Unlike many sports in which outcomes can often be predicted with a fair degree of certainty, football thrives on the unexpected. Whether it is an underdog team pulling off a stunning victory, a last-minute goal turning the tide, or a red card that shifts the balance of play, football’s inherent unpredictability is one of the reasons why it captivates millions around the world (5).

Given the great popularity of football worldwide, it has always been of interest to lovers of the sport to follow analyses, reviews, and predictions about the teams in various championships, as well as the natural inclination to try and guess which team will be crowned champion. Many people base their predictions on recent history, tradition, investments, players in the squad, or their passion for the teams.

Based on this principle, it is also natural to use science to make predictions about the results of different football tournaments. In particular, the field of statistics has proven to be very efficient in analyzing and understanding aspects of the game and making predictions about matches (6).

Bayesian inference makes it possible to incorporate aspects of the game that are often subjective, such as the opinions of experts, the political moment the club is going through, injuries to key players, or even a historical rivalry with an opponent, through appropriate prior distributions. Factors such as these can influence the final result of a match, and Bayesian prediction models can provide more satisfactory results when compared to other statistical methods.

In the present work, we compare 10 countable regression methods capable of predicting the results of the matches (number of goals) of the first division of the 2022 Brazilian Championship (Series A). This also enabled us to estimate each team’s attack–defense dynamic. We applied different aspects in the countable models, such as hierarchy, overdispersion, time-varying parameters, or informative priors. Since this study adopted the Bayesian approach, subjective probability based on informative prior distributions was reasonable, and this information comes from the 2021 Brazilian Championship, or through vague priors. Moreover, the inclusion of the squad value (monetary) effect was also tested. To the best of our knowledge, some elements of these learning structures have never been applied to Brazilian football statistical analysis, such as the dynamic Poisson model with zero inflation (this has not been applied to any match prediction in football championships). In the next section, we present the main statistical models that perform well in predicting sports results and have inspired us to choose the models used here.

The remainder of this paper is structured as follows. Section 2 provides an overview of models applied to football. Section 3 describes the approaches used in this work, the measures used to compare models, and the proposed Shiny framework. Section 4 provides statistical results and the interpretation of the models. Section 5 gives the conclusion and directions for future work.

## 2 The review of statistical methods in football

Various methods for quantifying football match performance have been explored in the literature, with Poisson models frequently employed to predict goal outcomes. For example, Araújo et al. (7) utilized a Poisson distribution to analyze the 2014 Brazilian Championship, while Olivieri Filho et al. (8) treated goal scoring as independent events, also using a Bayesian approach to assess team performance in the 2012–2013 English League. Other studies, such as those by Koopman and Lit (9), incorporated temporal dynamics into their predictions through dynamic models, employing bivariate Poisson distributions. In addition, methods such as zero-modified Poisson distributions and zero-inflated models have been developed to address issues of over- or under-dispersion, particularly in cases of many matches ending with zero goals (10). These advanced methodologies take into account various factors, including team rankings and match venues, to improve the accuracy of goal predictions in football.

Despite quantifying the attack–defense performance as the number of goals, incorporating match variation (goals scored and conceded) can be probabilistically modeled as the difference in goals per game. The sports literature considers this difference to be a Skellam distribution. For example, Karlis and Ntzoufras (11) propose a Bayesian approach to this distribution to model the goal difference between two teams rather than focusing on goals scored independently. The authors also make interesting extensions to this model, such as proposing an inflation of zeros, which in the case of the Skellam distribution means that both teams scored the same number of goals in the match, and correlating the attacking and defensive strengths of each team. Another example of applying the dynamic Skellam distribution to predict football results is discussed in Koopman et al. (12). The discrete Weibull distribution has also been used to predict football scores (13). Furthermore, Boshnakov et al. (14) present a forecasting model using the Weibull inter-arrival-times-based count process and also carry out bivariate modeling using a copula to produce a bivariate distribution for the number of goals scored by the home and away teams in a match. Bäcklund and Nils (15), in contrast, present an approach using a model based on the Negative Binomial distribution, comparing their results with those obtained from a Poisson model.

Many authors use Bayesian inference to adjust models for predicting football matches because it can incorporate prior information that can add quality to the predictions. For example, Suzuki et al. (16) used a Bayesian approach to predict the 2006 FIFA World Cup results. An exciting device used in this article is the incorporation of FIFA’s ranking as part of the prior distribution and expert opinions (guesses) about the participating teams. The authors use the de Finetti measure to assess the quality of the model, which produced satisfactory results, correctly predicting 57.81% of the matches in the competition. Salazar et al. (17) also proposed incorporating the results already observed during the World Cup as weights for the model’s subsequent predictions, being inversely proportional to the time elapsed when the match took place. In addition, the weights were also associated with expert opinions.

Moreover, from a Bayesian point of view, some studies consider a hierarchical model structure in which the parameters are estimated at different levels. Baio and Blangiardo (18) used a Bayesian hierarchical model for predicting Italian league matches for the 2007–2008 season, but they flag the problem of over-shrinkage, which for this model in the context of predicting football matches, can lead to underestimation or overestimation of the performance of some teams in the analyzed league. To solve this problem, the authors proposed creating more complex structures for the parameters, adding different classes of prior distributions according to the different levels of the teams. Egidi and Gabry (19) explored the application of Bayesian hierarchical models to predict the individual performance of soccer players. In addition to identifying important factors in the evaluation of this performance, based on evaluations provided by a popular Italian fantasy football game, these models were validated through graphical posterior predictive checks and provided out-of-sample predictions for the second half of the season. This approach is interesting because it escapes the usual application in the context of football, namely, mainly focusing on predicting match results.

Scholtes and Karakuş (20) also explored a hierarchical Bayesian approach, studying the influence of player or positional factors in predicting the probability of a shot resulting in a goal, measured by the expected goals (xG) metric. This provided an interesting look at advanced individual statistics that help build a more in-depth analysis of football matches. Another example is Blanco et al. (3), who used two Bayesian Poisson model structures to predict a team’s attack–defense performance in the 2020 Chilean Championship (comparing hierarchical and non-hierarchical models). They compared and assessed the quality of the models, using the leave-one-out cross-validation (LOOCV) metric. The models showed similar performance, leading the authors to opt for the non-hierarchical model due to its greater simplicity.

## 3 Materials and methods

In this paper, we have assumed that the goals scored by the home and away teams are independent and follow a Poisson distribution. This assumption is very common in studies that make predictions about football matches; see, e.g., Araújo et al. (7) and Olivieri Filho et al. (8). Furthermore, it can be seen that in the case of the 2022 Brazilian Championship, the choice of the Poisson distribution is justified by the fact that the average number of goals scored is very similar to its variance for both the home and away teams (mean of 1.41 and variance of 1.40 for the home team, and mean of 0.98 and variance of 0.79 for the away team).

This work analyzed the performance of teams in the 2022 Brazilian Championship Series A. In total, 20 teams played in the tournament, which had a total of 38 rounds, with 10 matches each round, resulting in a total of 380 matches. Every team confronted each other twice (once as the home team and the other as the visiting team). Five different types of Poisson models were tested, combined with and without hierarchy: (i) non-hierarchical (fixed-effect) Poisson regression, (ii) hierarchical Poisson (random effect with two levels), (iii) Poisson with zero inflation, (iv) dynamic Poisson, and (v) a dynamic Poisson model with zero inflation.

These two first statistical models were initially proposed by Blanco et al. (3). The first used a non-hierarchical structure in which the parameters were estimated for each team independently, while the second had a hierarchical structure, in which the matches were defined in the first level and the teams were in the second. Subsequently, in this work, the Poisson distribution with zero inflation was incorporated in the analysis, as proposed by Lambert (21), to consider the number of matches in which one or both teams do not score any goals higher than expected. Finally, dynamic models were adopted for the non-hierarchical and zero-inflated Poisson (ZIP) models, in which the attack, defense, and home-field factor parameters were updated every round.

**Figure 1:**
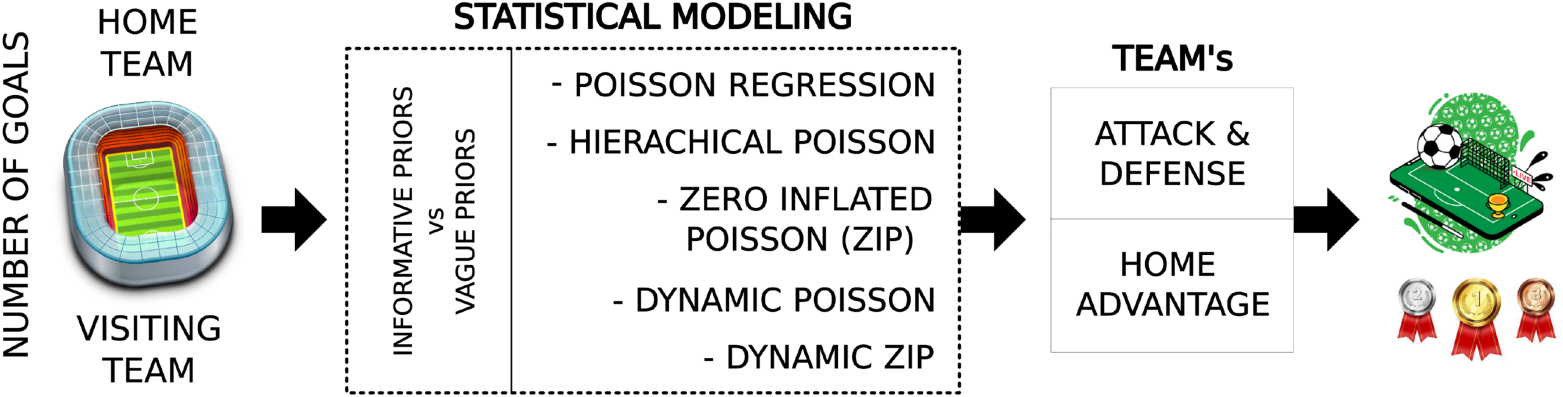
Visual methodology. Based on the number of goals (response variable), each team’s attack and defense is estimated through a statistical learning model.

Vague prior and informative prior distributions (explained below in this section) were also considered. The informative prior distribution incorporated attack and defense information from the previous seasons of the Brazilian Championship. Moreover, the squad value effect was considered, which refers to the monetary value of the team’s squad at the time of the championship match. Figure 1 summarizes the adopted methodology for estimates of each team’s attack and defense of the studied Brazilian Championship.

For all the models, predictions were made for the final 80 matches of the championship, i.e., the first 300 matches were used as training for testing the predictive power for matches 301–380, assessing the quality of the proposed models. The de Finetti measure and the percentage of correct predictions for each model were the forecast quality metrics. In addition, the LOOCV metric was used to compare the goodness of fit. Monte Carlo simulations estimated the results of each match in the competition, thus quantifying the probabilities of each team reaching certain stages of the tournament.

### 3.1 The theoretical models

In the context of football, we can consider that the number of goals scored by the gth team as the home team $\left(Y_{g1,t}\right)$ and as the visiting team $\left(Y_{g2,t}\right)$, in a match in round t, are independent and follow a Poisson distribution with parameter $\theta_{gi,t}$, for g=1,2,…,n (number of teams, n=20) and t=1,2,…,T (total of rounds, T=38 matches in the league, 19 as hosts then g1, and 19 as visitors then g2). Thus, $\theta_{g1,t}$ and $\theta_{g2,t}$ represent the average number of goals scored by the gth team in the tth round, the former as the home team and the latter as the visiting/away team.

In a Poisson regression model, Y is associated with independent variables x, in the form: $\theta =E[Y|x]=e^{x^{⊤}\beta }$. The number of goals scored by the home team is independent of the number of goals scored by the visiting team, i.e., $Y_{g1,t}\text{⫫}Y_{g2,t}$, then the home process mean is explained by the independent variables $x_{g1}=\{\text{``Attack home-team''},\text{``Defense visit-team''}\}$ and the visiting mean by $x_{g2}=\{\text{``Attack visit-team''},\text{``Defense home-team''}\}$. Thus, assuming the log-linear canonical link function, the models that were used to predict the number of goals scored were built as shown below.

#### 3.1.1 Non-hierarchical model

The non-hierarchical model defines the rate of goals ($\theta_{g1,t},\theta_{g2,t}$), that is, the expected number of goals per match, as follows:

(1) $$\log\;\left(\theta_{g1,t}\right)=\beta_{\mathrm{home}}+\beta_{1(g1)}att_{\mathrm{home}}-\beta_{2(g2)}def_{\mathrm{away}},$$

(2) $$\log\;\left(\theta_{g2,t}\right)=\beta_{3(g2)}att_{\mathrm{away}}-\beta_{4(g1)}def_{\mathrm{home}},$$

where $\beta_{\mathrm{home}}$ represents the home factor, att represents the attack of each team, def represents each team’s defense, $\beta_{1(g1)}$ represent the attacking strength and $\beta_{4(g1)}$ the defense of team g1 as the home team, and $\beta_{2(g2)}$ and $\beta_{3(g2)}$ represent the defense strength of the visiting/away team g2 (the opponent of team g1 in the tth round), respectively, for g1≠g2∈{1,2,…,20} teams.

An important feature of prior distributions is their variance, which governs how much spread or uncertainty is assumed in the parameters before the data are taken into account. High-variance priors are used when we want to allow the data to have a larger influence on the posterior distribution. In Bayesian inference, the concept of prior distribution plays a crucial role in modeling, and one of the main precautions regarding the choice of a prior distribution is to ensure that it is proper. A proper prior distribution has the property of being able to be normalized, i.e., it is a valid probability distribution; this is important because when calculating the posterior distribution, the normalization function must be finite for the posterior to be a valid probability distribution, thus enabling the inference process. Another important factor regarding the choice of a prior distribution is that even non-informative prior distributions have several advantages, such as modulating the influence of the data and facilitating computation and being able to provide flexibility when adjusting the model, especially in more complex structures. Thus, at first, the prior distributions of the parameters were defined as non-informative, as follows:

$$\begin{array}{rl} \beta_{\mathrm{home}} & ∼\text{Normal}\left(0,10^{4}\right),\;\text{and}\\ \beta_{\;j(g)} & ∼\text{Normal}\left(0,10^{4}\right), \end{array}$$

for j=1,2,3,4 and g={1,2,…,20} teams.

#### 3.1.2 Hierarchical model

The hierarchical model has the same likelihood as the first model, but now the attack and defense parameters do not depend on whether the team plays at home or not (that is, $\beta_{1(g1)}=\beta_{3(g2^{\prime })}\text{ and }\beta_{2(g2^{\prime })}=\beta_{4(g1)}$), and have a common structure between the teams:

$$\begin{array}{rl} \beta_{\mathrm{att}(g)} & ∼\text{Normal}\left(\mu_{\mathrm{att}},\sigma_{\mathrm{att}}^{2}\right),\\ \beta_{\mathrm{def}(g)} & ∼\text{Normal}\left(\mu_{\mathrm{def}},\sigma_{\mathrm{def}}^{2}\right), \end{array}$$

where g=1,2,…,20 teams, and the hyperparameters have the following non-informative prior distributions:

$$\begin{array}{rl} \mu_{\mathrm{att}} & ∼\text{Normal}\left(0,10^{4}\right),\;\sigma_{\mathrm{att}}^{2}∼\text{Gamma}\left(0.1,0.1\right),\\ \mu_{\mathrm{def}} & ∼\text{Normal}\left(0,10^{4}\right),\;\sigma_{\mathrm{def}}^{2}∼\text{Gamma}\left(0.1,0.1\right). \end{array}$$

In other words, in the non-hierarchical model, the attack and defense parameters are estimated independently. In contrast, in the hierarchical model, each team’s attack and defense parameters are described as an overall league average $(\mu_{\mathrm{att}}$ and $\mu_{\mathrm{def}})$, plus a random error with variance $\sigma_{\mathrm{att}}^{2}$ and $\sigma_{\mathrm{def}}^{2}$, respectively. Below in this article, when this structure incorporates the time-varying parameters, it will not matter if a team is playing at home or visiting because the attack (or defense) will be dynamic. But, before this, another recurrent case can be noticed whenever an excess of zeros (or zero inflation) occurs, which will be discussed in the following section.

#### 3.1.3 Zero-inflated Poisson model

In football, there is a considerable chance that a match will end without a goal being scored. For example, in the 2022 Brazilian Championship, approximately 29% of the time, one of the teams ended the match without scoring. Therefore, a ZIP model was fitted, as it should be used when the proportion of zeros in the data is higher than would be expected in a conventional Poisson model. ZIP is a mixture model that assumes a parameter ω as the probability of excessive zeros and a Poisson distribution with probability (1−ω), so its likelihood terms can be written as (21)

$$\begin{array}{rl} P\left(Y_{\left(g,t\right)}=0|\omega_{\left(g,t\right)},\theta_{\left(g,t\right)}\right) & =\omega_{\left(g,t\right)}+(1-\omega_{\left(g,t\right)})\;e^{-\theta_{\left(g,t\right)}},\\ P\left(Y_{\left(g,t\right)}=y_{\left(g,t\right)}|\omega_{\left(g,t\right)},\theta_{\left(g,t\right)}\right) & =(1-\omega_{\left(g,t\right)})\frac{e^{-\theta_{\left(g,t\right)}}{\theta_{\left(g,t\right)}}^{y_{\left(g,t\right)}}}{y_{\left(g,t\right)}!},\;\text{ for }y_{\left(g,t\right)}>0, \end{array}$$

where g=1,2,…,20 (number of teams) and t=1,2,…,19 (number of matches as host or visitor in the championship). In the context of predicting football match results, the proportion of matches in the previous season of the Brazilian Championship that ended with team g not scoring a goal was used as the prior average for the parameter $\omega_{g}$. Moreover, as in the non-hierarchical model, the parameters $\beta_{\;j(g)}$ were estimated, as in Equation 1 and in Equation 2, for j=1,2,3,4.

#### 3.1.4 Dynamic models

Another consideration of this work was to apply a dynamic character to the non-hierarchical and ZIP models. In a competition such as the Brazilian Championship, which occurs over several months, countless events can occur and result in a team’s improvement or deterioration in the competition, such as changes of coaches, the management of the squad to compete in other parallel competitions, player injuries, and the team’s natural evolution of performance. In other words, it is reasonable to think that a team’s attacking or defensive strength at time t differs from its strength at time t+h. Dynamic models allow these parameters to be updated over a certain period, and in this context, the parameters will be varied over the last eight rounds to capture changes in team performance throughout the predicted matches.

From the viewpoint of Bayesian inference, this variation will occur through the prior and posterior distributions of the parameters, i.e., the posterior distribution of the parameter at time t will be the prior distribution of this same parameter at time t+1, and so on; and for time t=1, a prior distribution can be freely specified.

Therefore, the teams’ attacking and defending parameters and the home factor are variable over time, and we can define the model used as follows:

$$\begin{array}{rl} \log\;\left(\theta_{g,t}\right) & =\beta_{\mathrm{home}(t)}+\beta_{1(g,t)}att_{\mathrm{home}}-\beta_{2(g^{\prime },t)}def_{\mathrm{away}},\\ \log\;\left(\theta_{g^{\prime },t}\right) & =\beta_{3(g^{\prime },t)}att_{\mathrm{away}}-\beta_{4(g,t)}def_{\mathrm{home}}, \end{array}$$

where the vague prior distributions, for the non-hierarchical dynamic Poisson model, were defined as

$$\begin{array}{rl} \beta_{\mathrm{home}(t=1)} & ∼\text{Normal}\left(0,10^{4}\right),\\ \beta_{\mathrm{home}(t)} & ∼\text{Normal}\left(\beta_{\mathrm{home}(t-1)},10^{-1}\right),\;\text{for}\;t=\{2,\ldots ,38\},\\ \beta_{i(g,t=1)} & ∼\text{Normal}\left(0,10^{4}\right),\;\text{for}\;i=\{1,\ldots ,4\},\mathrm{and}\;g=\{1,\ldots ,20\}\;\text{teams, and}\\ \beta_{i(g,t)} & ∼\text{Normal}\left(\beta_{i(g,t-1)},10^{-1}\right),\;\text{for}\;t=\{2,\ldots ,38\}. \end{array}$$

In the dynamic ZIP model,

$$\begin{array}{rl} & P\left(Y_{g,t}=0|\omega_{g,t},\theta_{g,t}\right)=\omega_{g,t}+(1-\omega_{g,t})\;e^{-\theta_{g,t}},\\ & P\left(Y_{g,t}=y_{g,t}|\omega_{g,t},\theta_{g,t}\right)=(1-\omega_{g,t})\frac{e^{-\theta_{g,t}}{\theta_{g,t}}^{y_{g,t}}}{y_{g,t}!},\;\text{for }y_{g,t}>0, \end{array}$$

and considering informative priors (based on the 2021 Brazilian Championship), the hyperparameters (in t=1) were

$$\begin{array}{rl} \beta_{\mathrm{home}(t=1)} & ∼\text{Normal}\left(0.3,0.15^{2}\right),\\ \beta_{i(g,t=1)} & ∼\text{Normal}\left(0,0.5^{2}\right),\text{for}\;i=\{1,\ldots ,4\},\;\mathrm{and}\;g=\{1,\ldots ,20\}\;\text{teams, and}\\ \;\theta_{g1,t=1} & ∼\text{Normal}\left(0.2263,10^{-1}\right),\\ \theta_{g2,t=1} & ∼\text{Normal}\left(0.3447,10^{-1}\right). \end{array}$$

### 3.2 Attack–defense prior information

Prior distributions represent information on the parameters before observing the data and are differently defined (22). In the first part of this study, these distributions were non-informative, but later, the non-hierarchical and hierarchical models were fitted to the 2021 Brazilian Championship. Then, it was possible to assume the posterior distribution of the attack and defense parameters in the 2021 season as the prior distribution of these parameters in the 2022 season. It is worth remembering that, according to the Brazilian League system, four teams are relegated to the second division and replaced by another four teams each year. Therefore, non-informative prior distributions were considered for the teams that were not in the first division in 2021.

Table 1 shows the averages of the teams’ attack and defense prior parameters. It can be seen that teams such as Atlético-MG (CAM) and Flamengo (FLA) have higher averages, while Juventude (JUV) and Cuiabá (CUI) have lower values. This is explained by the performance of these teams in the 2021 season. All acronyms are detailed in Appendix A.

**Table 1:** Prior averages of attack and defense parameters based on the application of the non-hierarchical and hierarchical models to the 2021 Brazilian Championship (we took the averages of the posterior distributions calculated for each participating team, thus, the performance of the teams in 2021 served as prior information for modeling the 2022 championship) and the team’s position (Pos.) in the 2021 season.

Team (Pos.)	Attack	Defense	Team (Pos.)	Attack	Defense
CAM (1)	0.5119	0.2689	CEA (11)	0.0156	0.0849
FLA (2)	0.5833	0.1464	INT (12)	0.1470	−0.0257
PAL (3)	0.4017	−0.0793	SAO (13)	−0.2198	0.1059
FOR (4)	0.1036	−0.0837	CAP (14)	0.0445	−0.0665
COR (5)	0.0420	0.1471	CUI (15)	−0.1577	0.1343
BRA (6)	0.3548	−0.1446	JUV (16)	−0.0902	−0.0744
FLU (7)	−0.0761	0.0839	BOT	0	0
AME (8)	0.0117	0.1015	GOI	0	0
ACG (9)	−0.2185	0.1149	CTB	0	0
SAN (10)	−0.1240	0.0530	AVA	0	0

The prior variance for these parameters was set at 0.1229, a value based on the observed posterior variances for the model applied in 2021, which changed very little for each team except for the teams coming from the second division [Botafogo (BOT), Goiás (GOI), Coritiba (CTB), and Avaí (AVA)], for which a non-informative prior was given as before.

### 3.3 Squad (monetary) prior information

In football, several factors can influence the outcome of a match. One of them is the squad value of the teams involved in the match. This information is often considered in analyses and guesses, as clubs with more expensive players are expected to achieve better results, especially in competitions that are played on a straight-points basis such as the Brazilian Championship. This variable was, therefore, used in the adjusted models.

Table 2 shows the squad values, in millions of euros, for the teams in the 2022 Brazilian Championship. These values were obtained from the Transfermarkt website (https://www.transfermarkt.com.br/campeonato-brasileiro-serie-a/marktwerteverein/wettbewerb/BRA1). Palmeiras (PAL), Flamengo (FLA), and Atlético-MG (CAM) have the most expensive squads, while Ceará (CEA), Juventude (JUV), and Avaí (AVA) have the cheapest ones. The squad value was included in the models as a covariate.

**Table 2:** Squad values, in millions of euros, for the teams in the 2022 Brazilian Championship Series A.

Team	Squad value	Team	Squad value
PAL	161.10	BOT	46.13
FLA	156.35	FOR	30.30
CAM	113.25	ACG	19.75
COR	85.75	CTB	19.13
BRA	81.23	AME	18.95
INT	78.70	CUI	18.75
SAO	76.70	GOI	15.83
CAP	67.20	CEA	15.50
SAN	65.15	JUV	15.25
FLU	58.60	AVA	14.23

Source: Transfermarkt.

### 3.4 Codes and reproducibility of analysis

This paper uses data from the 2022 Brazilian first division season. The results of the matches were obtained from: https://www.football-data.co.uk/brazil.php.

For those interested in replicating the presented applications, the datasets and R codes built to fit the models can be found at: https://github.com/GabrielRibeiro211/Modelo-ZIP-Dinamico-Campeonato-Brasileiro.

Since applying the countable regression models results in many combinations of football matches, there is a physical limitation to being able to show all the results in this article. Therefore, an online framework containing all possible results was created in a Shiny app. This framework is an open-result interactive tool, and is available at https://gyek75-gabriel-ribeiro.shinyapps.io/Campeonato_BR.

## 4 Results

The models described in the previous section were applied to data from the 2022 Brazilian Championship Series A. For each one of the 4,000 iterations, the number of goals predicted for the home team was compared with the number predicted for the away team, so that if the former was higher than the latter, it was considered a win for the home team; if it was lower, it was considered a loss and if they were the same, a draw. In this way, the probability of the home team winning was calculated as the proportion of this result in all 4,000 iterations. The probabilities of a draw and win for the away team are obtained following the same reasoning.

For each model, predictions were made for the last 80 matches in the league; that is, the first 300 matches were used as the training set for the models and the last 80 as the test. It was considered that the model was correct in the prediction when the outcome with the highest predicted probability was the same as that observed in the championship. For example, if the probability of the home team winning was 55% and the observed result was 2×1 for the home team, the model was considered correct because the home team won the match. However, this measure is not highly recommended for assessing the model’s predictive capacity because a high probability does not mean that an event is sure to occur, just like a low probability does not mean that an event is certain not to occur (8). We also used the de Finetti measure, commonly used in the literature to measure the quality of sports predictions, and the LOOCV metric to compare the Bayesian models.

### 4.1 Uncertainty analysis

**Figure 2:**
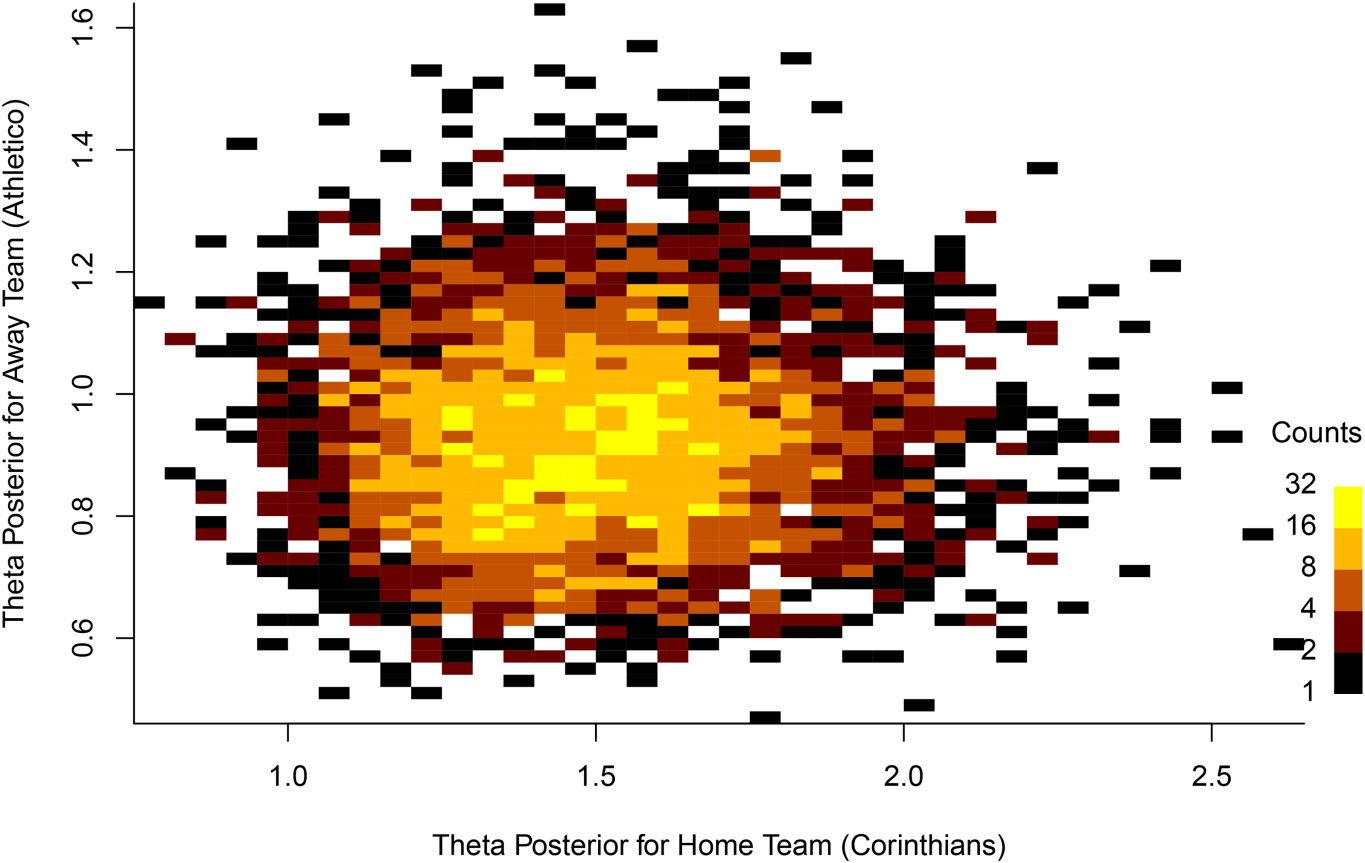
Posterior distribution of θ for Corinthians × Athletico-PR.

**Figure 3:**
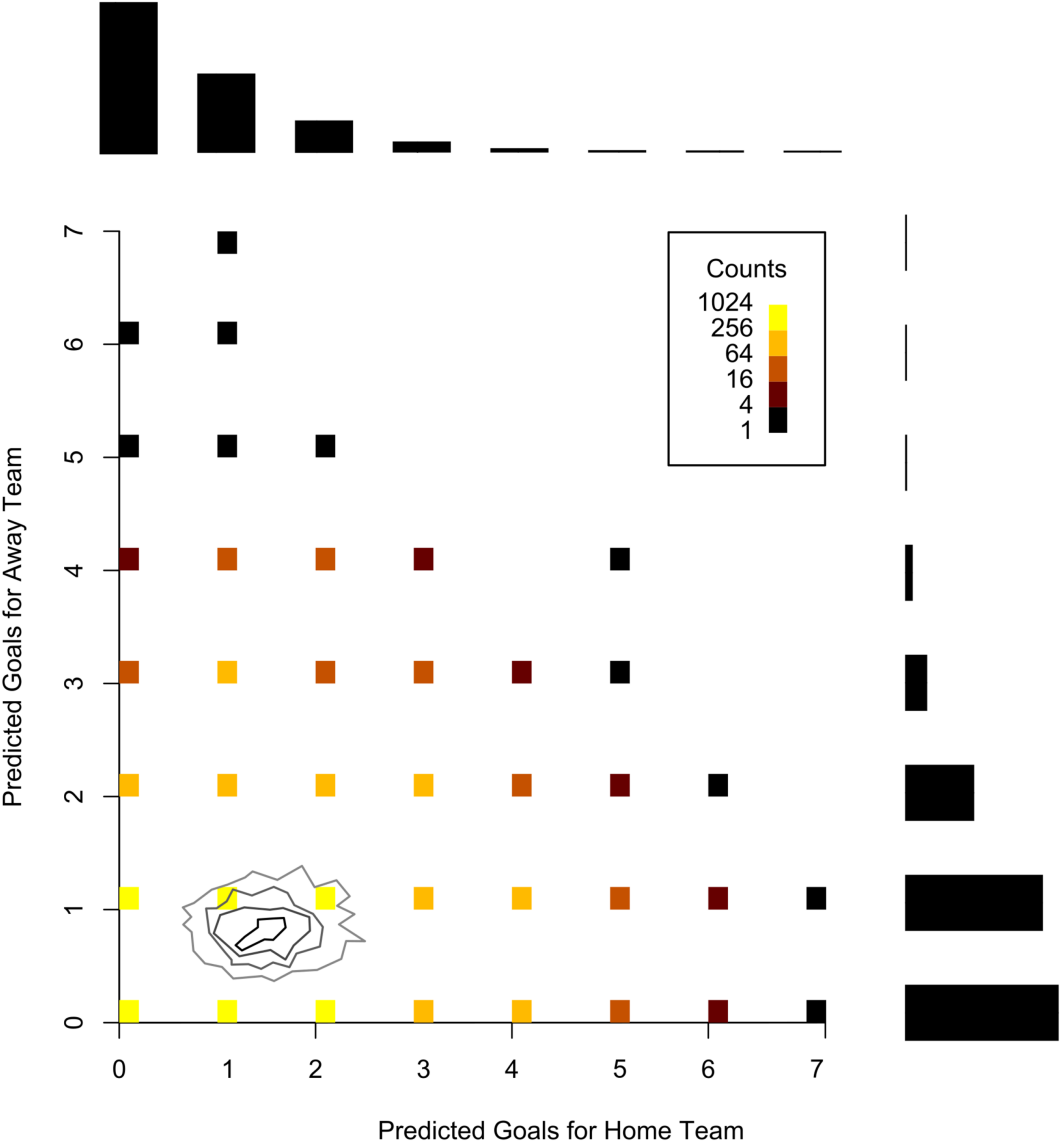
Distribution of predicted goals for Corinthians (home) × Athletico-PR (away).

Uncertainty quantification is a critical aspect of Bayesian analysis, as it provides a structured way to measure and incorporate uncertainty in a model’s parameters and predictions. In this context, uncertainty is explicitly accounted for in the model, rather than being ignored or reduced to a point estimate. Therefore, this makes Bayesian methods particularly powerful for understanding the range of possible outcomes, rather than just providing a single “best guess” for a parameter or prediction. In Bayesian analysis, we use credible intervals [(also known as highest posterior density intervals (HPDIs)]. In general, for the last 80 league matches we used to assess the predictive performance of the models, the 95% credibility interval of the average number of goals scored for the home teams in these matches ranged from 0 to 5, with an average of 1.57, while for the away teams, the estimates for this parameter ranged from 0 to 4, with an average of 1.08, indicating the advantage of playing as the home team. By using this type of analysis in each match, we can provide a more in-depth examination of the difference in strength between the teams, whether in attack or defense. To illustrate the uncertainty quantification regarding θ posterior values, Figure 2 shows the posterior distribution of θ for a specific league match, considering the *x*-axis for the home team and the *y*-axis for the away team. In this graph, it is possible to observe the parameter variation for both teams and the trend toward higher values for the home team. Figure 3 shows the distribution of the predicted goals based on the estimated parameters for this match. Figures 6 and 10 also help visualize and understand the variation in the estimated parameters.

### 4.2 Comparing the models

The following models were adjusted and compared:

• **Poisson regression**: Non-hierarchical (NH) model with non-informative prior distributions (called Simple NH);
• **Hierarchical Poisson regression**: Hierarchical (H) model with non-informative prior distributions (Simple H);
• **Previous season priors NH**: Non-hierarchical model with prior distributions referring to the previous season (2021) of the championship (Priors NH);
• **Previous season priors H**: Hierarchical model with prior distributions referring to the previous season (2021) of the championship (Priors H);
• **Squad information NH**: Non-hierarchical model with prior distributions referring to the previous season (2021) of the championship and with the squad value as a covariate (Squad NH);
• **Squad information H**: Hierarchical model with prior distributions referring to the previous season (2021) of the championship and with the squad value as a covariate (Squad H);
• **Dynamic Poisson regression**: Dynamic non-hierarchical model with prior distributions referring to the previous season (2021) of the championship and with the squad value as a covariate (Dynamic);
• **Dynamic Poisson with home-parameter prior**: Dynamic non-hierarchical model with non-informative prior distributions for the attack and defense parameters, but with an informative prior for the home factor parameter and the squad value as a covariate (Dynamic Home);
• **ZIP**: The ZIP model with non-informative prior distributions for the attack and defense parameters, but with an informative prior for the home factor parameter and the squad value as a covariate (ZIP);
• **Dynamic ZIP**: Dynamic ZIP model with non-informative prior distributions for the attack and defense parameters, but with an informative prior for the home factor parameter and the squad value variable (Dynamic ZIP).

Table 3 shows the number of correct predictions in the last 80 matches in the league and the de Finetti measure obtained for each fitted model. It can be seen that the simple NH model had the highest number of hits, correctly predicting the winner of 45 of the 80 matches, while the dynamic ZIP model had the lowest de Finetti measure. It should be remembered that, according to this measure, models with values lower than 2/3 can be considered to have good predictive quality, and the lower the value, the greater their predictive capacity (23). It is important to note that these models had a very similar performance in the training base (first 300 matches) compared to the test results.

**Table 3:** The number of correct predictions in the last 80 matches and the de Finetti measure for the fitted models.

Model	Correct predictions	de Finetti measure
Simple NH	**45**	0.6089
Simple H	44	0.6191
Priors NH	41	0.6280
Priors H	44	0.6184
Squad NH	40	0.6298
Squad H	40	0.6270
Dynamic	34	0.6544
Dynamic home	42	0.6018
ZIP	39	0.6041
Dynamic ZIP	40	**0.5988**

The values in bold indicate the best result in each metric.

In general, the models obtained similar results, successfully capturing the teams’ attacking and defensive strengths and generating predictions that make sense, given the clubs’ performances in the league. The fact that the dynamic ZIP model obtained the best de Finetti measure implies that this model generated more reliable predictions than others, even the NH model, which recorded the highest number of hits. Consider, for example, that one model provides probabilities of 40%, 30%, and 30%, while another model gives probabilities of 80%, 10%, and 10% for the home team winning, drawing, and losing, respectively. Given that the home team won, both models were right, but the second was clearly more accurate in its prediction, and this is exactly what the de Finetti measure reflects. Therefore, it is important to note that it is not correct to associate events with a high probability to a certainty of occurrence or events with a low probability to a certainty of non-occurrence. The aim in this type of work should not be to state that a given forecast is correct or not, but rather to construct a metric for the set of forecasts (23).

It is also important to note that accuracy values of around 50% at first glance may seem to indicate a low predictive performance. However, values like these end up being quite common when it comes to modeling the probabilities of victory, draw, or defeat of the teams in football match predictions [such as the models proposed by Santana et al. (5) and Araújo et al. (7), which obtained de Finetti measures of 0.64 and 0.56 and were correct for 40% and 50% of match results, respectively]. There are some factors that can explain this phenomenon. For example, football matches tend to be greatly influenced by events that are often difficult or even impossible to quantify satisfactorily, such as an injury or sending-off of an important player, adverse weather conditions, refereeing errors, and the natural variation in the performance of players and teams from one game to the next, in some cases due to psychological or motivational factors. These and many other aspects of the game cannot be directly taken into predictive models. Another aggravating factor, especially in stronger leagues, is the presence of similarity between the teams, which makes it difficult to define a favorite team to win the game, even among the opinions of expert football analysts. Therefore, predictive models that seek to predict football match results face great complexity due to the stochastic and dynamic nature of the sport. Despite this, predictive analysis is still useful for providing general insights into probabilities but it is important to understand its limitations in the face of football’s unpredictability.

For the best models, according to their hits and the de Finetti measure, their predictive posterior distribution accuracy was compared using LOOCV, with the R software function *loo_compare*(⋅). Table 4 shows the results of this function, considering the simple NH, dynamic home, ZIP, and dynamic ZIP models. The “ELPD diff” is the difference in ${\text{ELPD}}_{\text{LOOCV}}$ between the model with the largest ${\text{ELPD}}_{\text{LOOCV}}$ and the other models so that the preferred model always receives the value of zero, and the remaining models receive the negative values. The “SE diff” is the standard error in the difference in ${\text{ELPD}}_{\text{LOOCV}}$ (24). According to the LOOCV, the best-fitted model was the dynamic ZIP, which also had the lowest de Finetti measure. Thus, by the definition of the de Finetti measure, the dynamic ZIP model provides higher probabilities of the event occurring than, for example, the NH model (which had the largest number of hits), when both models were wrong or right. Therefore, in the next section, we will present some dynamic ZIP model prediction results.

**Table 4:** Model comparison by the LOOCV metric.

Model	ELPD diff	SE diff
Dynamic ZIP	0.0	0.0
Dynamic Home	−1.0	0.9
ZIP	−1.6	1.0
Simple NH	−8.2	2.8

Another reason why the dynamic ZIP model achieved better results compared to the others is its ability to monitor the evolution of each team as the rounds elapsed, which is of great value in a championship of such a long duration. In addition, the inflation of zeros made it possible to model the occurrence of games in which at least one of the teams failed to score a goal. Finally, this model is advantageous because, despite its greater complexity and the greater number of parameters to be estimated, its interpretation remains similar to others.

### 4.3 Dynamic ZIP model

The dynamic ZIP model was fitted by setting the parameter for the proportion of excess zeros to ω=(0.2263,0.3447), which is related to the proportion of matches in which the home and away teams did not score in the 2021 Brazilian Championship, respectively. In addition, non-informative prior distributions were considered for the teams’ attack and defense parameters, but for the home factor parameter, a Normal(0.3,0.15) prior distribution was assigned, based on the performance of the home teams in past seasons. The squad value covariate was also incorporated into the model.

**Figure 4:**
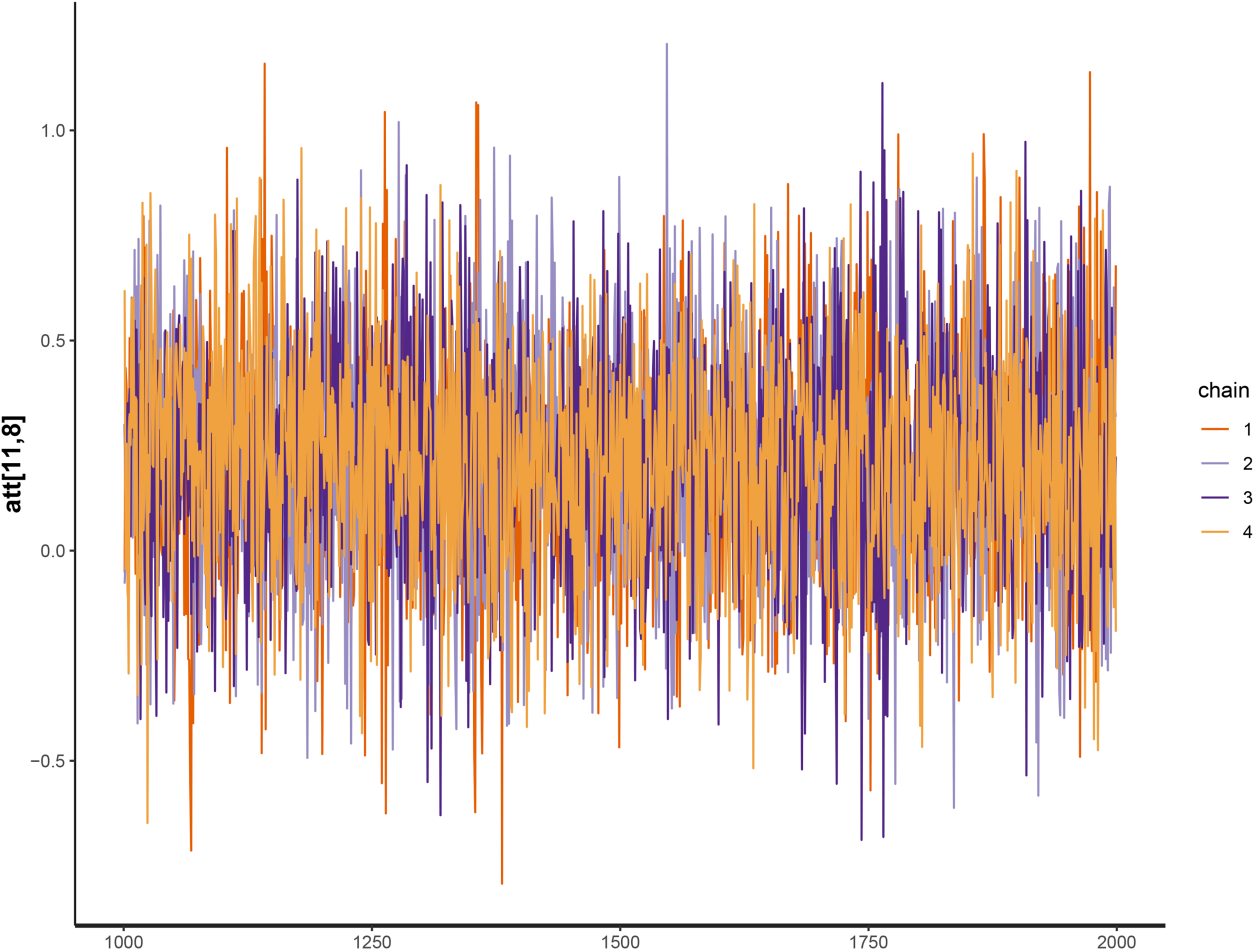
Trace plot of the attack parameter (Palmeiras).

**Figure 5:**
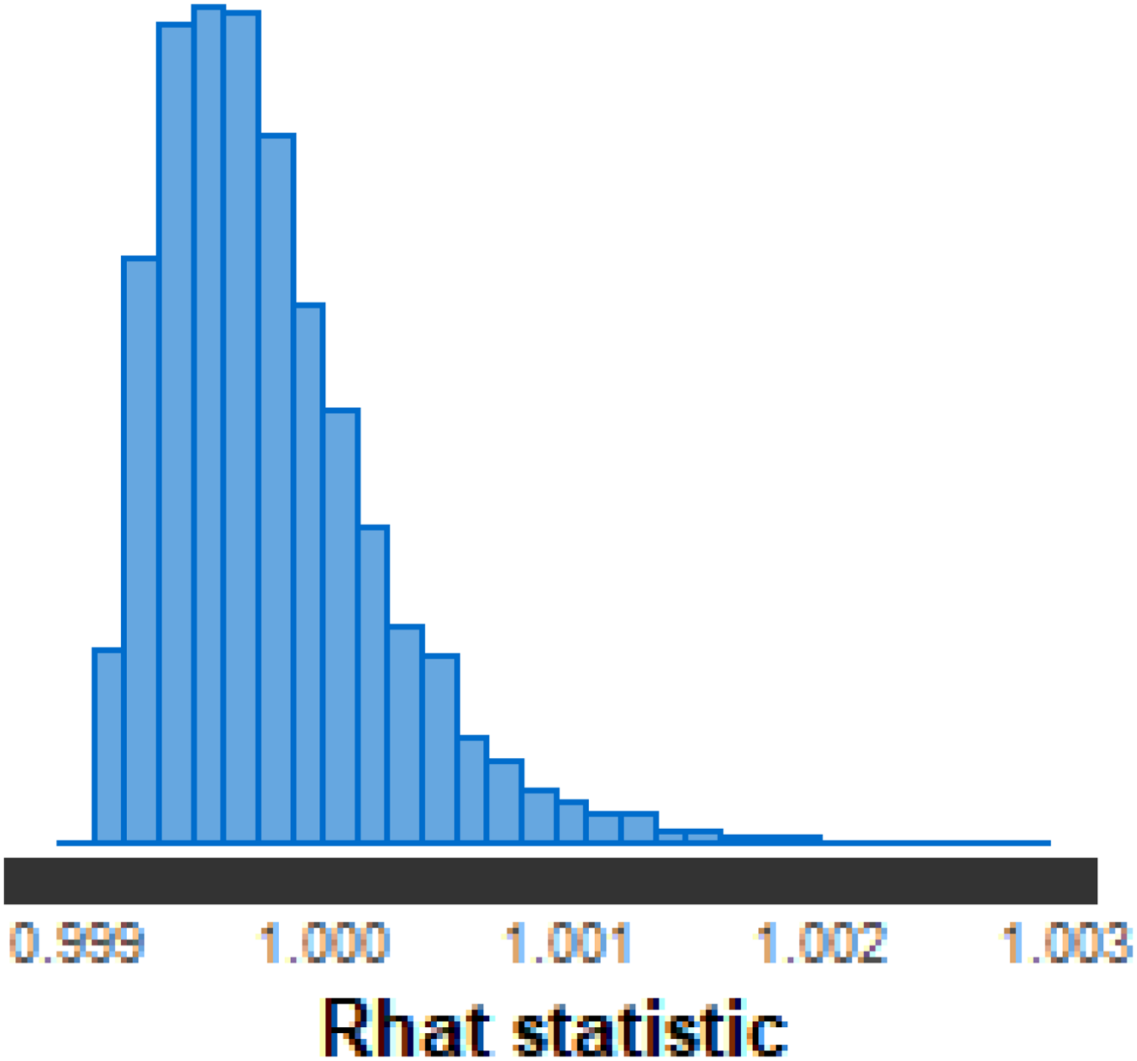
Shrinkage factor $\hat{R}$.

When analyzing the convergence of the Markov chains, there were indications that these chains converged for all of the model’s parameters. For example, Figure 4 shows the trace plot of Palmeiras’ attack parameter chains at time 8, where the initial 1,000 iterations (burn-in) have been discarded. It can be seen that the four chains show similar behavior around the same values, indicating that convergence has been reached. In Figure 5, it can be seen that the shrinkage factor $\hat{R}$ of the home factor parameter is distributed around 1, which indicates convergence of the Markov chains for this parameter.

**Figure 6:**
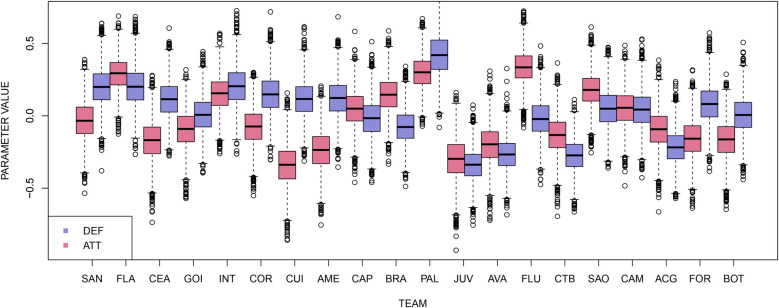
Boxplots of the teams’ attacking and defensive strengths in the 38th round.

As there is evidence that the distributions have reached convergence, it is possible to analyze them. Figure 6 shows the boxplot of each team’s attacking and defensive strengths in the 38th round. Palmeiras (PAL), the team winning the competition, has the best defensive strength and the second-best attacking strength. Juventude (JUV), on the other hand, has low values for both attack and defense, which is explained by the team’s poor performance and finishing at the bottom of the competition.

**Figure 7:**
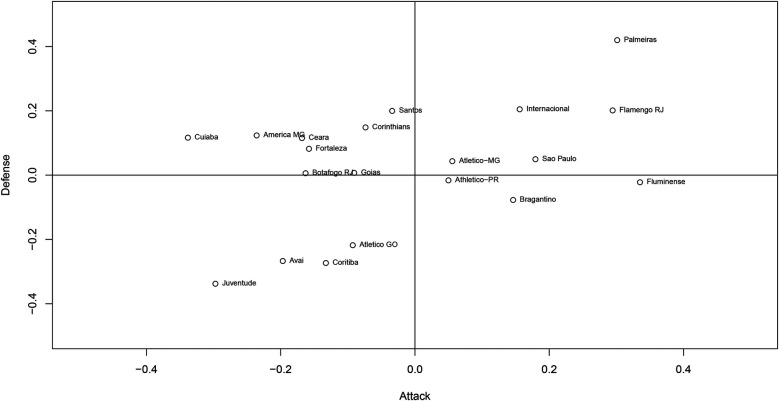
Relationship between the attacking and defensive strengths.

Figure 7 shows the relationship between the attacking and defensive parameters of the teams in the championship, where the horizontal axis shows attacking strength and the vertical axis defensive strength. Teams with better attacks tend to also have better defenses. We can immediately highlight two teams, Palmeiras and Juventude: while the champions, Palmeiras, had the best defense parameter and the second-best attack parameter, the last-placed Juventude had the worst defense parameter and the second-worst attack parameter, as seen before. The teams in the first quadrant can be considered to have good attack and good defense, such as Palmeiras, Flamengo, and Internacional. This performance is considered ideal, and unsurprisingly, most of the teams in this quadrant occupied the top positions in the league. In the second quadrant, there are teams with good defensive performance but poor attacks, such as Santos, Corinthians, and América-MG. In the third quadrant, there are teams with poor performance for both parameters, which also reflects the performance of these teams in the league, and three of the teams were relegated to the second division that year. Finally, in the fourth quadrant, some teams had a good attack (Fluminense had the highest attack parameter of all the teams) but a poor defensive performance.

**Figure 8:**
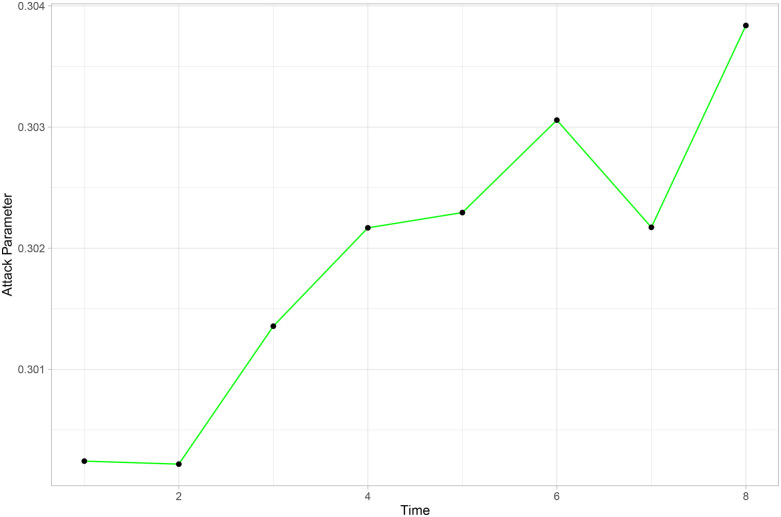
Evolution of Palmeiras’ attacking parameter.

To illustrate the dynamic nature of the model, Figure 8 shows the evolution of Palmeiras’ attack parameter over the rounds considered in the adjustment. It can be seen as an expected behavior for a good team; that is, in general, the attacking parameter increases over time.

**Figure 9:**
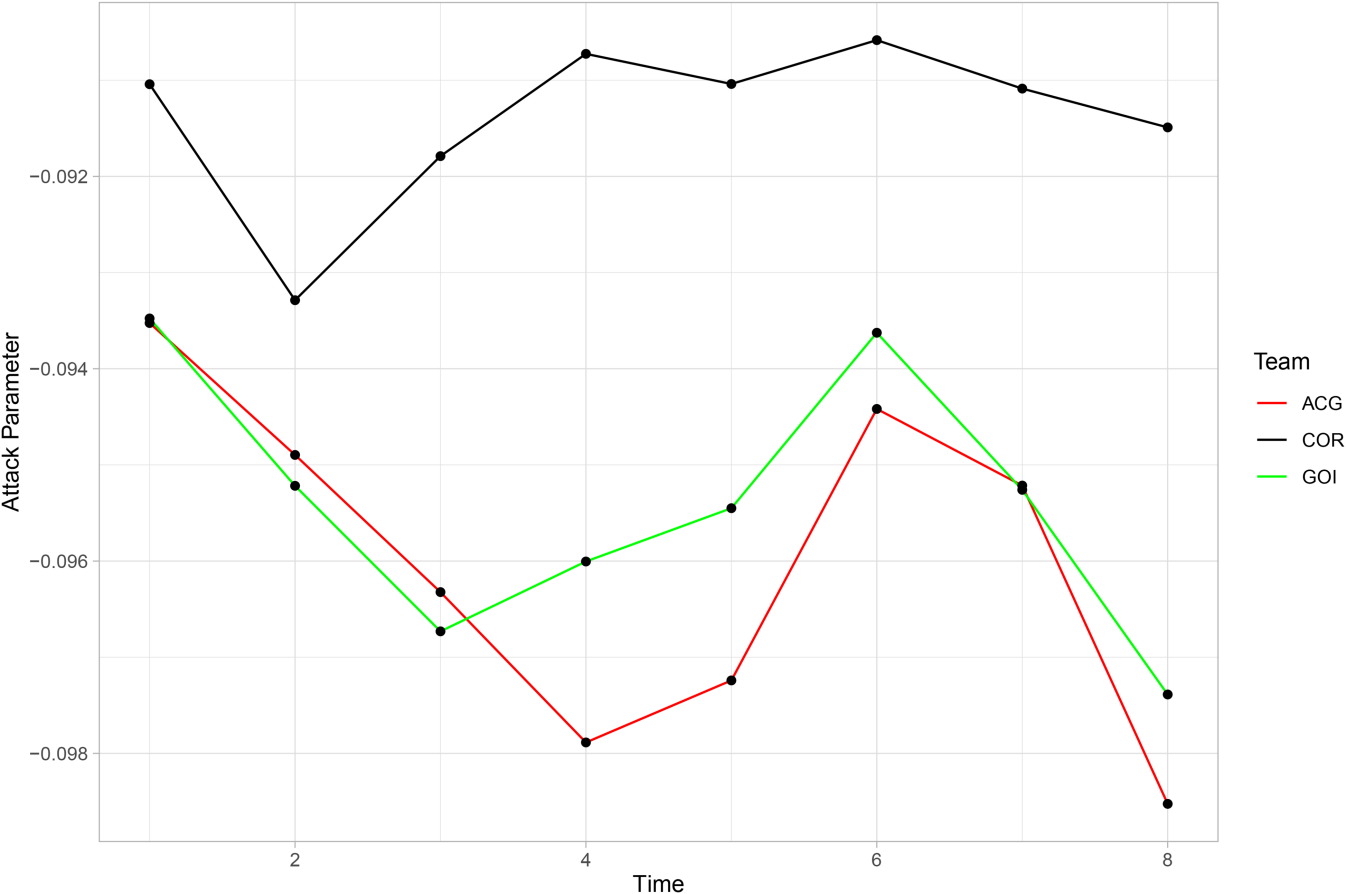
Comparison of the evolution of the attacking parameters of Corinthians, Atlético-GO, and Goiás.

However, Figure 9 shows different patterns for three teams that had similar attacking parameters: Corinthians (COR), Atlético-GO (ACG), and Goiás (GOI). In the first round considered for prediction, the parameters of Atlético-GO (in red) and Goiás (in green) were the same, but in rounds 2 and 3, Atlético-GO had higher parameters, reversing the relationship in the following rounds. Corinthians’ attack parameter remained unchanged and was higher than the other two in every round.

**Figure 10:**
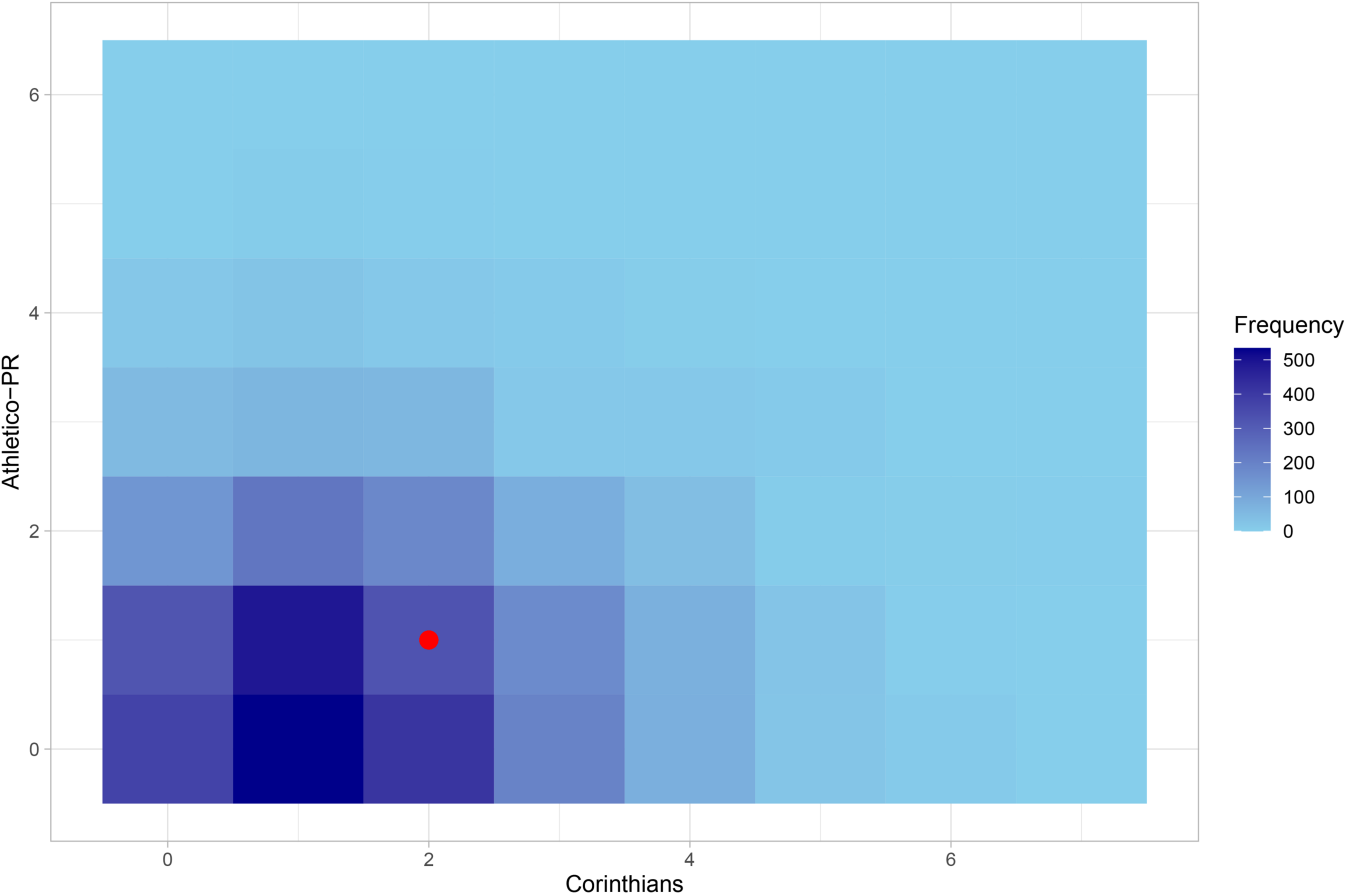
Heatmap of the forecast for Corinthians vs. Athletico-PR.

To show how the predictions for each match work, Figure 10 exhibits a heatmap of the predictions for the result of the Corinthians vs. Athletico-PR match, which took place in round 31 of the championship. The areas in darker blue represent the most likely scores according to the model, which are 1×0, 1×1, 2×0, and 2×1; and the red dot represents the result observed in this match, which was 2×1, with victory for Corinthians. Therefore, it can be considered that this model provided a good prediction for this match, as the highest probability was given for the home team (Corinthians) to win.

**Figure 11:**
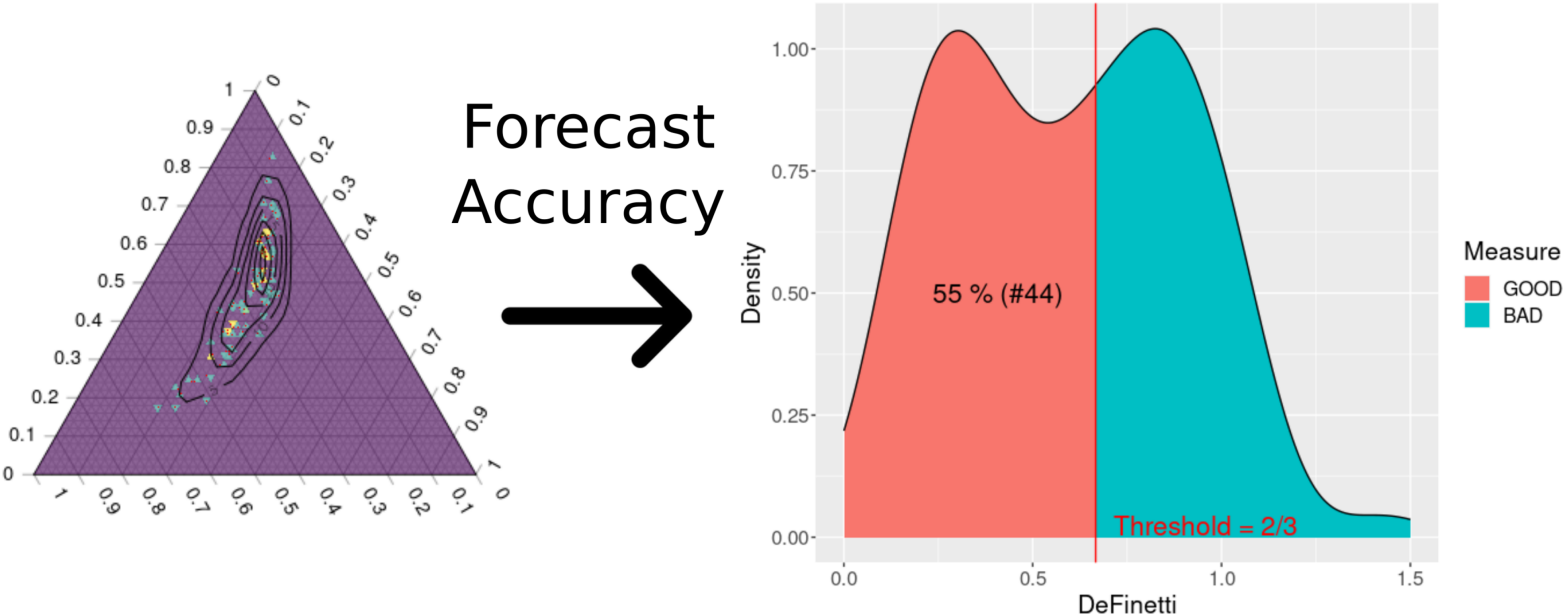
The dynamic ZIP model’s de Finetti simplex transformed into a forecast accuracy measure for the 2022 Brazilian Championship Series A.

It was also observed that the squad value variable positively influenced the expected number of goals for each team, with the average parameter being approximately 0.14. Finally, to check whether the predictions of the dynamic ZIP model work for the Brazilian Championship, the same model was also fitted for the previous three seasons of the competition. Figure 11 shows the dynamic ZIP model’s simplex, and its forecast accuracy measure was summarized as a histogram density.

Table 5 shows the number of hits in the 80 matches predicted for each season and the associated average de Finetti measure for these seasons. It can be seen that the model achieved similar performances over these years, except for the 2020 season, in which the model obtained the worst result. However, it is worth remembering that this season underwent some changes due to the COVID-19 pandemic that devastated the country at the time, such as the lack of supporters in the stadiums and a high number of players missing due to positive diagnoses of the disease. Therefore, the dynamic ZIP model fits well with the data from the Brazilian Championship in general.

**Table 5:** Adjustment of the dynamic ZIP model to different seasons of the Brazilian Championship.

Season	Correct predictions	de Finetti measure
2022	40	0.5988
2021	40	0.5984
2020	38	0.6359
2019	40	0.5856

## 5 Conclusions and future works

In this study, different Bayesian models were compared in order to predict the results of football matches in the 2022 Brazilian Championship Series A. Models based on the Poisson distribution were fitted, with hierarchical and non-hierarchical structures, zero inflation, and a dynamic character for the non-hierarchical and ZIP models.

According to the comparison metrics used, the model proposed here (dynamic Poisson model with zero inflation) was preferred for predicting the results of the Brazilian Championship matches. It is worth highlighting that, to the best of our knowledge, this model has not been applied to football championships before. Its average de Finetti measure was 0.5988 and 50% of the predicted results were correct. Therefore, the dynamic ZIP model obtained satisfactory results when compared to other applications in the Brazilian football data, such as the models proposed by Santana et al. (5) and Araújo et al. (7), which obtained de Finetti measures of 0.64 and 0.56 and were correct for 40% and 50% of match results, respectively.

In addition, it is worth noting that these models were applied to a league with points in hand, in which each team plays against all the others, at home and away. However, there are various other forms of competition in football leagues, which may require other forms of adjustment to predict their matches. Therefore, the best model in one context may not work well in others.

For future works, some extensions to the approaches presented here may be considered, such as the inclusion of more covariates that can influence the final result of a match; the application of a dynamic hierarchical model; and the use of other distributions, e.g., the Skellam distribution, used by Karlis and Ntzoufras (11) and Koopman et al. (12), or the hurdle version of the Poisson model (as an alternative to the Poisson model with zero inflation). It is also interesting to assess whether the dynamic ZIP model continues to have the best performance in other national points championships or in championships with other forms of competition.

## Data Availability

The datasets presented in this study can be found in online repositories. The names of the repository/repositories and accession number(s) can be found below: https://github.com/GabrielRibeiro211/Modelo-ZIP-Dinamico-Campeonato-Brasileiro.

## Appendix A: acronyms

**Table:**

ACG	Atlético clube goianiense
AME	América futebol clube – Minas gerais
AVA	Avaí futebol clube
BOT	Botafogo de futebol e regatas
BRA	Red bull bragantino
CAM	Clube atlético mineiro
CAP	Club athletico paranaense
CEA	Ceará sporting club
COR	Sport club corinthians paulista
CTB	Coritiba foot ball club
CUI	Cuiabá esporte clube
FLA	Clube de regatas flamengo
FLU	Fluminense football club
FOR	Fortaleza esporte clube
GOI	Goiás esporte clube
INT	Sport club internacional
JUV	Esporte clube juventude
PAL	Sociedade esportiva palmeiras
SAN	Santos futebol clube
SAO	São Paulo futebol clube
