# Supplementary material for: A Bayesian approach to predict performance in football: a case study
Source: Front Sports Act Living. 2025 Mar 14;7:1486928. doi: 10.3389/fspor.2025.1486928 (PMC11949986; doi:10.3389/fspor.2025.1486928)
Supplement: Supplementary file 1 [file Datasheet1.pdf]

Supplementary Material

This supplementary material discusses some concepts about the estimation method used (Section 1) and elucidates the performance measures applied in this work (Section 2).

1 THE APPROXIMATE BAYESIAN COMPUTATIONAL METHOD

The Hamiltonian Monte Carlo (HMC) approach is a well-established approximate Bayesian computational method to estimate the model parameters with easy convergence. According to Betancourt (2017), HMC is a Markov chain Monte Carlo (MCMC) method that, to make the chain traverse the parametric space in a more functional way, adds an auxiliary variable called *momentum* (ρ). Thus, the parametric space is explored through partial derivatives of Hamilton's equation, converging much faster.

In many applications, the density of the auxiliary variable $\boldsymbol{\rho}$ is defined as a multivariate (or d-variate) Normal that does not depend on θ:

$$\boldsymbol{\rho} \sim \text{Normal}_d\left(\mathbf{0}, \Sigma\right),$$

where Σ (covariance matrix) acts as an Euclidean metric of rotation and scale of the distribution of interest, allowing greater chain flexibility when traversing its parametric space.

The joint density $p(\rho, \theta)$ is defined by

$$H(\rho, \theta) = -\log\left(p(\rho, \theta)\right) = -\log\left(p(\rho \mid \theta)\right) - \log\left(p(\theta)\right) = T(\rho, \theta) + V(\theta),$$

where T is called *kinetic energy* and V is called *potential energy*.

The state transitions are made using the Metropolis acceptance method. First, a $\rho \sim \text{Normal}_d\left(\mathbf{0}, \Sigma\right)$ is generated, then the ρ and θ values of the joint distribution $p(\rho, \theta)$ are updated via Hamilton's equations:

$$\begin{aligned}
\frac{d\theta}{dt} &= \frac{\partial H}{\partial \rho} = \frac{\partial T}{\partial \rho}, \\
\frac{d\rho}{dt} &= -\frac{\partial H}{\partial \theta} = -\frac{\partial T}{\partial \theta} - \frac{\partial V}{\partial \theta}.
\end{aligned}$$

Thus, considering ϵ as a time interval between simulations, a new $\rho \sim \text{Normal}_d\left(\mathbf{0}, \Sigma\right)$ is generated and the ρ and θ values are updated in the following scheme:

$$\rho \leftarrow \rho - \frac{\epsilon}{2}\frac{\partial V}{\partial \theta},$$

$$\theta \leftarrow \theta + \epsilon \Sigma \rho,$$

$$\rho \leftarrow \rho - \frac{\epsilon}{2}\frac{\partial V}{\partial \theta}.$$

By repeating the above steps L times, the simulation will result in the values (ρ^*, θ^*), which will be accepted with probability $\alpha = \min\{1, \exp\{H(\rho, \theta) - H(\rho^*, \theta^*)\}\}$.

To apply the models to the Brazilian Championship matches, we used the `rstan` package (Stan Development Team, 2023) from the R software, which is a package for Bayesian inference using the C++ language. RStan fits Bayesian models primarily using the No-U-Turn Sampler (NUTS) algorithm, and it is possible to obtain the outputs, including posterior inferences and intermediate quantiles, such as log-posterior density assessments and their gradients. To fit the models specified above, the command $stan(\cdot)$ was used, with $chains = 4$ (which defines the number of Markov chains), $iter = 2000$ (which defines the number of iterations made in each chain), and $warmup = iter/2$ (which defines how many iterations will be used as a warm-up or burn-in, so they will not be used to make inference). Thus, the inference process was carried out with a sample of size 4,000 from the posterior distribution.

2 PERFORMANCE MEASURES

This section discusses the adopted performance measurements that guided choosing the best model for explainability of each team's attack-defense power as a home team or visiting team. The DeFinetti measure informs the quality of the model's prediction as a minimal necessary utility information (Nau, 2001), which serves as a forecast accuracy metric. LOOCV helps generalize the fitted models by splitting the dataset into K-folds and checking if the best-of-fitting is valid for each block (or just a matter of random luck). Finally, the Gelman-Rubin diagnosis shows elements towards the convergence of the random samples to the target posterior distributions and the convergence of posterior means to the target mean through two properties: i) stationarity, and ii) mixing samples (Du et al., 2022).

2.1 DeFinetti Measure

The DeFinetti measure, proposed by De Finetti (1972), was used to assess the quality of the model's predictions. It considers the squared Euclidean distance between the predictions and the observed results, defined in a *simplex* contained in $\mathbb{R}^3$ (de Araújo et al., 2015). In other words, as in a football match, there are three possible outcomes: i) the Home Team Wins (HW), ii) the Away Team Wins (AW), and iii) a Tie Game (TG), then the simplex is defined as follows:

$$S = \{(\mathbb{P}(\text{'HW'}), \mathbb{P}(\text{'AW'}), \mathbb{P}(\text{'TG'})) \in \mathbb{R}^3 : \mathbb{P}(\text{'HW'}) + \mathbb{P}(\text{'AW'}) + \mathbb{P}(\text{'TG'}) = 1,$$
$$\mathbb{P}(\text{'HW'}) \geq 0, \mathbb{P}(\text{'AW'}) \geq 0, \mathbb{P}(\text{'TG'}) \geq 0\},$$

where $\mathbb{P}(\text{'HW'})$, $\mathbb{P}(\text{'AW'})$, and $\mathbb{P}(\text{'TG'})$ represent the predicted probabilities of the home team winning, the away team winning, and drawing, respectively, associated with the vertices $(1, 0, 0)$, $(0, 1, 0)$, and $(0, 0, 1)$. Figure S1 shows the representation of one triple (e.g., game #01) in the simplex.

The DeFinetti measure is the calculation (summary) of the ternary as prediction according to the observed result:

$$(\mathbb{P}(\text{'HW'}) - 1)^2 + (\mathbb{P}(\text{'AW'}) - 0)^2 + (\mathbb{P}(\text{'TG'}) - 0)^2 \,,$$

if the home team wins the match;

$$(\mathbb{P}(\text{'HW'}) - 0)^2 + (\mathbb{P}(\text{'AW'}) - 1)^2 + (\mathbb{P}(\text{'TG'}) - 0)^2 \,,$$

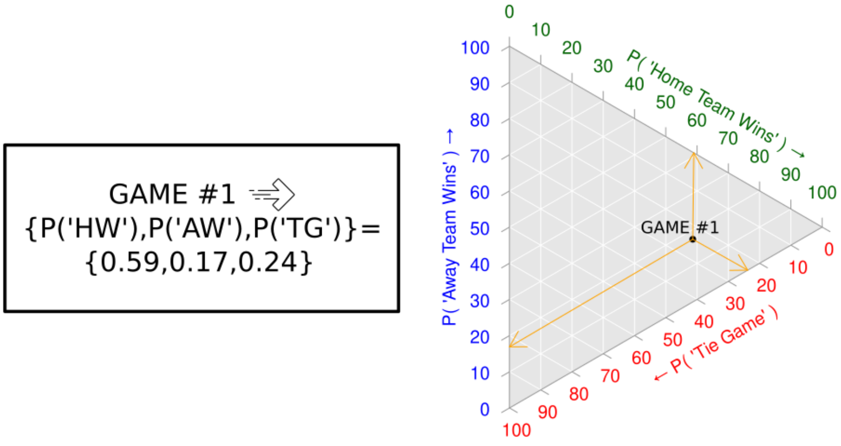

Figure S1. Illustration of the DeFinetti distance in a simplex as a ternary plot (%). The Home Team Wins (HW) is represented as the vertex $(1, 0, 0)$ in green color, the Away Team Wins (AW) as the vertex $(0, 1, 0)$ in blue color, and a Tie Game (TG) as the vertex $(0, 0, 1)$ in red color.

if the home team loses (away team wins) the match; and

$$(\mathbb{P}(\text{‘HW’}) - 0)^2 + (\mathbb{P}(\text{‘AW’}) - 0)^2 + (\mathbb{P}(\text{‘TG’}) - 1)^2 ,$$

if the match ends in a draw (tie game). For instance, let us consider the match #01’s estimated ternary, $\{\mathbb{P}(\text{‘HW’}), \mathbb{P}(\text{‘AW’}), \mathbb{P}(\text{‘TG’})\} = \{0.59, 0.17, 0.24\}$. Then, if the home team wins, the DeFinetti measure would be ≈ 0.258; if the away team wins, but the adjusted model predicted only a $\mathbb{P}(\text{‘AW’}) = 0.24$, then the DeFinetti measure would be 0.95; and if a tie game is observed, this measure would be 1.09. Therefore, each match’s quality prediction (DeFinetti measure) is based on the adjusted model versus the match’s outcome.

If equal probabilities are assigned to each result, that is, if $\mathbb{P}(\text{‘HW’}) = \mathbb{P}(\text{‘AW’}) = \mathbb{P}(\text{‘TG’}) = 1/3$, the DeFinetti measure will be $2/3$ (for any observed scenarios). Therefore, any number greater than $2/3$ shows a poor quality of the ternary prediction towards the observed outcome. From this point of view, the match forecasting quality acceptance should be less than 2/3 (de Araújo et al., 2015).

2.2 Leave-One-Out Cross-Validation (LOOCV)

When two or more models are fitted, it is interesting to use comparison methods to see which fits the data better. In the Bayesian context, one of the most popular model comparison metrics is LOOCV, which measures the predictive accuracy of a sample unit considering all the others. This metric is constructed based on the predictive posterior distribution for a new sample $\tilde{x}$, defined as:

$$p(\tilde{x} \mid \boldsymbol{x}) = \int p(\tilde{x} \mid \theta)\, p(\theta \mid \boldsymbol{x})\, d\theta.$$

First, Vehtari et al. (2017) defined a measure of predictive accuracy for n data points taken one at a time, called the expected log pointwise predictive density (ELPD):

$$\text{ELPD} = \sum_{i=1}^{n} \int p_t\left(\tilde{x}_i\right) \log\left(p(\tilde{x}_i \mid \boldsymbol{x})\right) d\tilde{x}_i,$$

where $p_t\left(\tilde{x}_i\right)$ is the distribution representing the true generative process of the data for $\tilde{x}_i$. However, since this distribution is unknown, the LOOCV can be estimated approximately as:

$$\text{ELPD}_{\text{LOOCV}} = \sum_{i=1}^{n} \log\left(p\left(x_i \mid \boldsymbol{x}_{-i}\right)\right),$$

where $p\left(x_i \mid \boldsymbol{x}_{-i}\right) = \int p\left(x_i \mid \theta\right) p\left(\theta \mid \boldsymbol{x}_{-i}\right) d\theta$ is the predictive density of the i-th observation conditioned on the data without it.

In the R software, it is possible to compare Bayesian models using LOOCV by using the $loo\_compare(\cdot)$ function, where the model with the highest $\text{ELPD}_{\text{LOOCV}}$ always receives a value of zero in the comparison. The remaining models receive negative values (Vehtari et al., 2017).

2.3 Gelman-Rubin Diagnosis

The Gelman-Rubin (GR) diagnostic is one of the most popular methods for evaluating samples obtained from MCMC algorithms. The GR diagnostic depends on several chains starting from the initial points defined from a density overdispersed concerning the target density (Roy, 2020).

Gelman and Rubin (1992) define the method as constructing two estimators of the variance of X, where X follows the distribution of interest π. These estimators, defined from m parallel chains, are: W, the variance within each chain; and B, the variance between the chains, where:

$$W = \frac{1}{m}\sum_{i=1}^{m} s_i^2, \quad \text{with } s_i^2 = \frac{1}{n-1}\sum_{j=1}^{n}\left(\overline{x}_{ij} - \overline{x}_{i.}\right)^2,$$

and

$$B = \frac{n}{m-1}\sum_{i=1}^{m}\left(\overline{x}_{i.} - \overline{x}_{..}\right)^2.$$

The variance of X is then calculated as:

$$\widehat{\mathbb{V}\text{ar}}[X] = \frac{n}{n-1}W + \frac{1}{n}B.$$

Finally, these estimators are compared, making it possible to verify the existence of convergence of the Markov chains through the shrinkage factor:

$$\widehat{R} = \frac{\widehat{\mathbb{V}\text{ar}}[X]}{W}.$$

The shrinkage factor uses the same idea as an analysis of variance (ANOVA), in which the variance within each chain is compared with the variance between them. The closer the ratio between the variances is to 1, the greater the indication that the chains converge towards the distribution of interest.

REFERENCES

Betancourt, M. (2017). A conceptual introduction to hamiltonian monte carlo. *arXiv preprint arXiv:1701.02434*

de Araújo, C. T. P., Tavares, L., Alvares, L. G., Neto, F. L., and Suzuki, A. K. (2015). Modelagem estatística para a previsão de jogos de futebol: Uma aplicação no campeonato brasileiro de futebol 2014. *Revista da Estatística da Universidade Federal de Ouro Preto*

De Finetti, B. (1972). Probability, induction and statistics: The art of guessing

Du, H., Ke, Z., Jiang, G., and Huang, S. (2022). The performances of gelman-rubin and geweke's convergence diagnostics of monte carlo markov chains in bayesian analysis. *Journal of Behavioral Data Science* 2, 47–72

Gelman, A. and Rubin, D. B. (1992). Inference from iterative simulation using multiple sequences. *Statistical science* , 457–472

Nau, R. F. (2001). De finetti was right: probability does not exist. *Theory and Decision* 51, 89–124

Roy, V. (2020). Convergence diagnostics for markov chain monte carlo. *Annual Review of Statistics and Its Application* 7, 387–412

[Dataset] Stan Development Team (2023). RStan: the R interface to Stan. R package version 2.26.13

Vehtari, A., Gelman, A., and Gabry, J. (2017). Practical bayesian model evaluation using leave-one-out cross-validation and waic. *Statistics and computing* 27, 1413–1432
